# Ethical dilemma of identity disclosure faced by medical students in clinical clerkships: A nationwide multicenter study in China

**DOI:** 10.1371/journal.pone.0200335

**Published:** 2018-07-11

**Authors:** Yi Zhao, Yihan Cao, Lu Che, Qining Fu, Shuang Song, Bingbin Zhao, Shuo Zhang, Weiwen Zhang, Xiang Li, Stephanie Choi, Jun Zhao, Hanwen Zhang, Yunzhu Li, Haopeng Xu, Hui Pan

**Affiliations:** 1 Peking Union Medical College Hospital, Peking Union Medical College and Chinese Academy of Medical Sciences, Beijing, China; 2 Department of Anesthesiology, Peking Union Medical College Hospital, Peking Union Medical College and Chinese Academy of Medical Sciences, Beijing, China; 3 Department of Vascular Surgery, The First Affiliated Hospital of Chongqing Medical University, Chongqing, China; 4 Xiangya School of Medicine, Central South University, Changsha, China; 5 Tongji Medical College, Huazhong University of Science and Technology, Wuhan, China; 6 Harvard Medical School, Boston, the United States of America; 7 Department of Medical Education, Peking Union Medical College Hospital, Peking Union Medical College and Chinese Academy of Medical Sciences, Beijing, China; 8 West China Medical Center, Sichuan University, Chengdu, China; Brown University, UNITED STATES

## Abstract

**Objective:**

Medical students in China are currently facing a dilemma of whether to clarify their identity as students to patients. Further investigation is needed to support policy-making. The aim was to identify factors influencing medical students’ decision on whether or not to clarify their identity to patients and to examine the effects of their decision.

**Methods:**

The study was a cross-sectional nationwide multicenter survey consisting of 947 medical students. A self-designed questionnaire was composed of 19 structured questions investigating the present situation and participants’ perception of the ethical dilemma surrounding medical student identity. The questionnaires were distributed randomly in teaching hospitals affiliated with 13 medical schools across China from June 2015 to January 2016.

**Results:**

A total of 947 valid questionnaires were retrieved with a valid response rate of 83.7%. Most medical students (71.4%) tended to be ambiguous about their student identity in front of patients. The frequency of encountering distrust and patients’ or patient relatives’ refusal to allow students to perform procedures was significantly lower for students who explicitly stated their identity than for those who were ambiguous about their identity (p<0.001). Less experience in clinical rotations (<0.5 y/0.5–1 y, OR 2.7, 95% CI 1.7–4.3; <0.5 y/>1 y, OR 3.6, 95% CI 2.0–6.5), preceptors’ straightforward introduction of the students (OR 8.7, 95% CI 5.4–13.8) and students’ acknowledgment of patients’ right to know (OR 2.3, 95% CI 1.2–4.5) were related to students’ clear self-introduction to patients.

**Conclusion:**

It is beneficial for medical students to clearly explain their identity to patients in order to decrease patient distrust and prevent the refusal to have certain appropriate procedures performed. Several methods, including emphasizing the role of mentors, developing curriculum for medical students, and creating clear regulations and guidelines for revealing the identity of medical students on the healthcare team can help address and ideally resolve this ethical dilemma of identity disclosure.

## Introduction

Clinical clerkship, a stage when learners in medicine move from the classroom to the workplace of practicing physicians [1], is an essential component in the education of a future physician. Through exposure to daily patient management during clerkship, students gain essential hands-on experience in a practical clinical setting. Many medical students in China are now facing a rather common ethical dilemma of whether to clarify their role as medical students while interacting with patients.

On the one hand, medical students are supposed to actively interact with patients and be directly involved in patient care. Because medical students are not qualified to practice as doctors themselves, informing patients that medical students are involved in their medical care may make patients anxious [2] and increase the probability of refusal. On the other hand, patients’ autonomy and choice of health care providers should be respected and Chinese patients are becoming more aware of their right to give informed consent about medical student involvement in their care. When patients discover later that student involvement was concealed, they may feel deceived and may lose trust in doctors, which is detrimental because this trust is the core of doctor-patient relationships [2].

Meanwhile, the absence of laws defining the obligations and rights of medical students in clinical clerkship means that, to some extent, medical students do not actually have “a legal identity” in clinical practice, and this lack of legal support intensifies the dilemma. Therefore, the conflict between patients’ autonomy and the awkward, vague identity of medical students results in a dilemma of both medical education and legislation, leading to problems in daily medical care and teaching [3].

There is an ongoing discussion about student involvement in medical practice [4], and many studies have been carried out from the patient perspective. Generally, Asian patients exhibit lower levels of acceptance and comfort with respect to medical student teaching than Western patients do [5]. An observational study in Japan suggested that only one-fourth of participants accepted medical care by students, and the top reasons for refusal consisted of feeling anxious, taking extra time, fearing that the students lacked skills and knowledge, and perceiving medical students as unreliable [6]. Furthermore, student gender, the level of student involvement and the student’s training stage also influenced patients’ level of comfort with medical students [7,8]. Although many studies have focused on patient attitudes, few studies have focused on the attitudes of medical students, which is also a research gap in China.

This study looks into this issue from the perspective of medical students. Our aim is to identify the factors influencing students’ decision to clarify their roles or not and to examine the effects of their decision, which may guide future reform in teaching, student administration and related laws of clinical clerkship.

## Methods

### Study design

The study was a cross-sectional nationwide multicenter survey among 13 medical schools in 9 cities in China. Medical students in clinical clerkship were surveyed about their attitudes towards the identity dilemma and related issues in clinical practice. A self-designed questionnaire written in Chinese was used in the study. Confirmed by the Institute Review Board (IRB) of Peking Union Medical College Hospital, the study can be exempt from IRB review.

All thirteen medical schools are key public colleges in China that provide high-quality long-term (7-year or 8-year) clinical medicine education programs. All the teaching hospitals affiliated with these schools are Grade A tertiary hospitals with at least 500 beds located mainly in East and Southwest China. Medical students in these schools undergo clinical rotations for two years beginning in their 4^th^ to 5^th^ year with slight variation among the different schools.

### Questionnaire

The questionnaire (a translated English version is provided in S1 File) was composed of 19 structured questions and 1 open question divided into two parts. The first part addressed the demographic characteristics of the participants, including gender, medical school, time in clinical clerkship, whether clinical medicine was the first choice when applying for college, whether they had relatives who were doctors or nurses, and prior experience being hospitalized or accompanying an inpatient for personal reasons. The second part investigated three aspects of the issue of identity as well as their comments on this issue:

1. How supervisors introduce students to patients and how students introduce themselves to patients, specifically whether they stated the student’s role clearly (e.g., “we have a medical student on our team / I am a medical student on the team”), or ambiguously (e.g., “This is Dr. … / I am Dr. …”);
2. Adverse events encountered in patient interactions in the last six months, i.e. events that negatively affected bedside teaching, including distrust from patients, refusal to be allowed to perform medical procedures, and conflicts between patients and doctors;
3. Students’ subjective attitudes towards the following issues:

- Whether it is reasonable for patients to distrust medical students;
- Whether patients have every right to know the true identities of all team members involved in their medical care;
- Whether schools should train the tutors to cope with such issues;
- Whether their patients realize that it is routine for medical students to participate in medical care in teaching hospitals.

### Data collection

Data were collected from June 2015 to January 2016 by distributing paper questionnaires randomly in teaching hospitals affiliated with the 13 medical schools. Oral informed consent was obtained from every participant after explaining the purpose of the survey and noting that the data collected would be used only for academic purpose. Two operators inputted the data independently and excluded those with questions left blank. The questionnaires with falsified information as judged by the operators were also excluded. The authors had no access to information that could identify participants during and after the date collection.

### Statistical analysis

Students were divided into subgroups based on their answers to how they introduced themselves to patients. The differences between the subgroups were analyzed by the chi-square test or Fisher’s exact test when needed. Factors with significant differences (independent variables) were applied to bivariate logistic regression to explore the factors influencing students’ ways of introducing themselves (dependent variable). The significance level was set at 0.05. Statistical analysis was performed with SPSS 21.0.

## Results

### Demographic features

A total of 1300 questionnaires were issued and 1132 were retrieved. After screening, 947 questionnaires were valid with a valid response rate of 83.7%. A maximum of 87 and a minimum of 61 valid questionnaires were retrieved from each school. Students’ answers to the question are included in S1 Dataset.

Table 1 shows the demographic features of the investigated students. Of the 947 medical students, 560 (59.1%) were female. Students who had been in clinical rotation for less than 6 months, 6 months to 1 year and more than 1 year constituted 57.7%, 22.5% and 19.8% of the participants, respectively. Most students (88.5%) had selected clinical medicine as their first choice when applying for colleges. Nearly half (47.4%) of the surveyed students had one or more relatives who were doctors or nurses. A total of 67.2% had been hospitalized or had accompanied their relatives or friends in the hospital previously.

**Table 1:** Demographic characteristics of the 947 surveyed medical students.

Characteristics	No. (%)
Gender	
*Male*	387 (40.9)
*Female*	560 (59.1)
Clinical Experience	
*Less than 6 months*	546 (57.7)
*6 months to 1 year*	213 (22.5)
*More than 1 year*	188 (19.8)
Clinical medicine as first choice	
*Yes*	838 (88.5)
*No*	109 (11.5)
Relatives who are doctors or nurses	
*At least one*	449 (47.4)
*None*	498 (52.6)
Prior experience of being hospitalized or accompanying inpatients	
*Yes*	636 (67.2)
*No*	311 (32.8)

### Means of introduction and adverse events

59.0% of the students stated that their supervisors would disclose their identity to patients and emphasize that it is routine in teaching hospitals for students to be engaged as members of the medical team. On the other hand, 41.0% of the students reported that their supervisors would use the word “doctor” to introduce them, obscuring their true identity. From the perspective of students, 28.6% of them would clearly state their identity (e.g., “I am a medical student from … medical school”) and ask for permission to participate in patient care. The remaining 71.4% were ambiguous about their identity (e.g., introduced themselves with a statement such as “I am Dr. …”) and would not clearly explain that they were a student unless asked.

**Fig 1:**
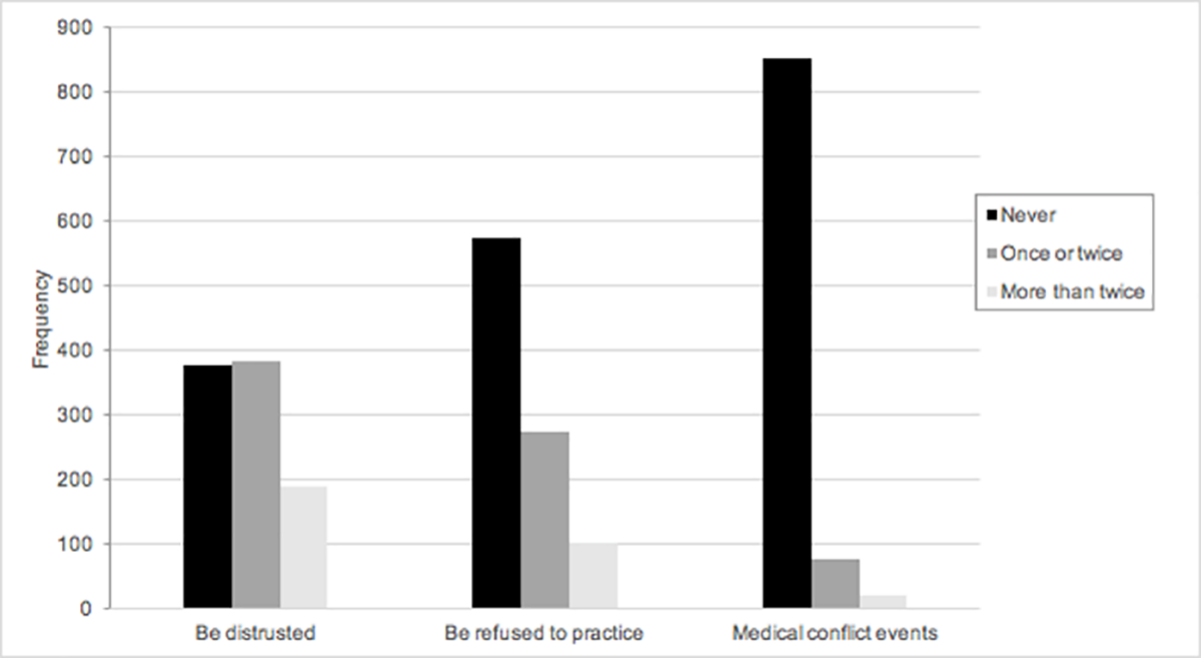
Adverse events that medical students had encountered in the past 6 months.

Fig 1 illustrates the frequency of adverse events encountered by medical students over the past six months. Specifically, 60.4% of the students reported being distrusted by the patients or their relatives at least once, and 33.2% had encountered this situation more than twice. A total of 39.5% of the students had encountered patients who refused to allow them to perform medical procedures, and 10.2% had experienced conflict events between patients and physicians due to their identity as a medical student.

Notably, the frequency of being distrusted by patients or their relatives was significantly lower for students who clearly stated their identity (55.4% never, 31.0% once or twice, 13.7% more than twice) than for those who spoke ambiguously (33.3% never, 44.1% once or twice, 22.6% more than twice) (p<0.001). With regard to the refusal to give permission to perform medical procedures, the frequency was also significantly lower when the identity of the students was addressed clearly rather than ambiguously (Table 2). However, the results revealed no significant difference was found in terms of conflict events between patients and physicians.

**Table 2:** Adverse events encountered by medical students according to how they introduce themselves to patients.

Adverse events	Frequency	Means of introduction by students		P
		Stated clearly (%)	Ambiguous (%)	
		271(100)	676(100)	
Distrust from patients	0	150(55.4)	225(33.3)	P<0.001
	1–2	84(31.0)	298(44.1)	
	≥3	37(13.7)	153(22.6)	
Patient refusal to give permission to perform medical procedures	0	197(72.7)	376(55.6)	P<0.001
	1–2	54(19.9)	219(32.4)	
	≥3	20(7.4)	81(12.0)	
Conflict event between patients and physicians	0	242(89.3)	608(89.8)	P = 0.235
	1–2	20(7.4)	57(8.4)	
	≥3	9(3.3)	11(1.6)	

### Factors influencing students’ means of introduction

Six factors, including gender, clinical experience, hospitalization experience, preceptors’ method of introducing students, students’ attitudes towards patients’ rights and whether related training for preceptors is necessary, showed significant differences between students who introduced themselves clearly and those who introduced themselves ambiguously (Table 3), and these factors were included in the bivariate logistic regression analysis as the possible factors influencing students’ means of introduction. Ultimately, three factors played a significant role including clinical experience, preceptors’ method of introduction and students’ attitudes towards patients’ right to know (Table 4). Students who had more experience in clinical rotation tended to be ambiguous with their identities (<0.5 y/0.5–1 y, OR = 2.7, 95% CI 1.7–4.3; <0.5 y/>1 y, OR = 3.6, 95% CI 2.0–6.5). If preceptors clearly introduced the identity of students, students themselves also tended to be clear about their identity (OR = 8.7, 95% CI 5.4–13.8). Students who thought that patients had the right to know the true identities of every member of their medical team were more likely to introduce themselves honestly and ask for permission from the patients before performing medical activities (OR = 2.3, 95% CI 1.2–4.5).

**Table 3:** Chi-square test of factors influencing how students introduce themselves to patients.

Items	P
Gender	P = 0.034
How long have you been directly involved in patients' diagnosis and treatment process in your clinical internship?	P<0.001
Was clinical medicine your first choice when applying for college?	P = 0.143
Do you have any family members who are also health care workers?	P = 0.430
Have you ever been hospitalized, or have you ever taken care of someone who was hospitalized?	P = 0.022
When your superiors/clinical preceptors introduce you to the patients and their families, how do they address you?	P<0.001
How do you feel about patients' distrust towards clinical interns?	P = 0.581
Do you feel that the patients have every right to know the true identities of all team members involved in their medical care?	P<0.001
Do you think that the school should train clinical preceptors to deal with such issues?	P = 0.008
Do you think that the patients realize that it is routine for medical students to participate in medical care in teaching hospitals?	P = 0.379

**Table 4:** Optimal bivariate logistic regression model for possible factors influencing how students introduce themselves to patients.

Variables	Logistic regression coefficient	OR	95% CI	P
Clinical experience (<0.5y / 0.5-1y)	1.0	2.7	1.7–4.3	<0.001
Clinical experience (<0.5y / >1y)	1.3	3.6	2.0–6.5	<0.001
Preceptors’ method of introduction (clear / ambiguous)	2.2	8.7	5.4–13.8	<0.001
Attitude towards patients’ right to know (yes / no)	0.8	2.3	1.2–4.5	0.011

### Means of introduction in different medical activities

Only 17.8% of the students thought it necessary to disclose their identity and obtain informed consent before history taking and physical examination. An even lower percentage (15.3%) of the students deemed it necessary when conducting noninvasive medical procedures such as dressing change. However, the proportion was remarkably higher when it came to invasive medical procedures, with 36.0% agreeing for low-risk procedures such as venous blood draws and 75.6% for high-risk ones such as bone marrow aspiration and lumber puncture. A significant difference was found in the means of introduction when students performed different types of medical procedures (P<0.001).

### Students’ attitudes towards issues related to the identity dilemma

Up to 75.9% of the students regarded distrust from patients as being not completely reasonable but understandable, while 21.2% deemed it fair and acceptable (S1 Table). Only 2.9% of the students thought the distrust stemmed from personal prejudice. Students’ attitudes towards this distrust had no relation with their gender, their clinical experience, whether they had relatives who were medical workers or their prior personal experience with hospitalization. Furthermore, 90.2% of the students agreed that patients had every right to know the true identities of all team members involved in their medical care, and 90% thought it necessary to provide related training for clinical tutors on how to address the issue of medical students’ identity. According to 40% of the students, patients did not realize that it was a routine for medical students to be involved in their medical care if they came to a teaching hospital.

## Discussion

Professional identity formation (PIF) is increasingly considered a major focus for medical education [9]. According to the theory, it is both an individualized and socialized process for a medical student to transform from a member of the lay public to an individual “thinking, acting and feeling like a physician”, during which “the characteristics, values, and norms of the medical profession are internalized” [10,11]. The transformation takes place in stages and it is difficult to skip stages, as individuals at a lower stage (e.g., medical students) are less developmentally advanced and cannot fully understand the issues faced by individuals at a higher stage (e.g., residents) [12]. Medical students are in the early stage of “imperial” in Kegan’s stages of identity formation, where they start to “pretend” to be physicians by observation and imitation, but are not fully recognized as physicians [13,14]. The ethical dilemma of identity disclosure is inevitably encountered by individuals in this stage universally [2], and handling it properly will ideally support successful acquisition of their new identity.

PIF includes developing medical ethics of a humanistic physician in addition to required competency-based education with one’s own unique quality and core values [15,16]. Honesty and respect for patients’ autonomy are basic requirements in medical ethics, and disclosure of student status is a direct reflection of these norms [17]. Deception in identity disclosure can lead to distrust from patients [2], as evidenced by our survey findings that disclosure of student identity to patients was associated with a lower frequency of adverse events in patient interactions. Previous research also showed that patient satisfaction in several aspects of care was improved after medical students were formally introduced [18]. While medical students may not always have permission from patients to be involved in their care, students can play various essential roles including taking a more in-depth history and at times providing essential details. Our study raises the ethical implications of paltering with student status and perceptions of this, both for students and for mentors. The need for awareness of a potential “cascade of small indignities” to prevent ethical erosion has been described [19].

The situation indicated by the results is concerning. Based on our research, most medical students preferred to conceal their true identity as students. This can be explained by the fact that the identity of a “doctor” rather than a “student” facilitates engagement in patient care [20]. This is further corroborated by the study conducted by Pallin and colleagues regarding the significant decrease in the rate of consent after informing patients of medical students’ participation [21]. However, the situation differed in regard to procedures with diverse risks. More medical students in our survey deemed it necessary to disclose their student identity and ask for permission when performing high-risk invasive procedures such as lumber puncture rather than other noninvasive or low-risk invasive procedures such as history taking or venous blood draws. From the perspective of patients, previous studies also found that only 7% of patients would permit medical students to perform lumber puncture for the first time at a tertiary medical center, whereas the rate of acceptance of venipuncture was 42% [22].

Socialization is a key element in the process of PIF, and role models and mentors have the most powerful influence on socialization [23]. Our findings were consistent with this, given that supervisors’ emphasis on students’ true identities in front of their patients was associated with a higher probability that their students would clearly introduce themselves and ask for permission prior to providing clinical care. Medical students transit from a layperson to professional by imitating and practicing what their role models or mentors do (and not imitating negative role models), which is how role models and mentors exert their influences [24]. Some behaviors can be unconscious, but have the same power. Therefore, role models and mentors should be explicit about what they are modeling, especially in issues related to medical ethics [24]. Nevertheless, not all mentors can correctly distinguish unethical practices and always act ethically [25]. Faculty development programs as well as institutional support and a system of evaluation are crucial to address the gap [26]. Appropriate attitudes towards this dilemma should become part of mentors’ identity and be reflected in observable behaviors [27].

Moral courage is needed within the practice of medicine and many medical educators believe that moral courage can be taught and strengthened [28]. The inclusion of reflective discussion on various dilemmas in practice and the need for moral courage within ethics and/or PIF curriculum can help address the issues our study raises. In addition to guided reflection, the integral role of relationships, formative feedback, and the creation of collaborative learning environments or communities of practice are core elements of such courses [16,29]. Our study found that medical students who recognized patients’ right to know were also more likely to clearly introduce themselves, suggesting that realizing the rights and obligations of both patients and medical students can motivate morally courageous behavior; thus, the curriculum should include such content. Clinicians’ (and trainees’) emotional responses to moral distress resulting from adverse events should also be addressed in the curriculum to help cultivate moral resilience, complementing traditional ethics education [30]. In line with the results of a U.S. survey [31], we found that students who had been in clinical rotation for a longer time tended to be more ambiguous about revealing their student identity to patients, suggesting that such education should be implemented during the entire clinical clerkship experience and be tailored appropriately for junior and senior students because they tend to think differently on the issue.

Legislation is another essential issue regarding the problem, as pointed out by over half of the surveyed students. In Britain, for example, the General Medical Council (GMC) determines the educational standards and contents for medical students, expounded in its several published documents including *Tomorrow’s Doctors* [32], *Good Medical Practice* [33] and *Medical students*: *Professional values and fitness to practice* [34]. The expected outcomes and practical procedures of medical school graduates are explicitly established in *Tomorrow’s Doctors*. This document also clarifies that medical students should follow the guidance of *Good Medical Practice* and that the medical school should consider whether it amounts to a fitness of practice concern if a student’s behavior falls below these expected levels [32–34]. These documents not only define students’ roles and responsibilities in clinical practice but also protect patients’ safety and support maintaining their trust in students to a maximum extent. We strongly recommend formal medical education legislation in China to legally address the issue of medical students’ identity disclosure within clinical care contexts and to set practical guidelines for students, supervisors and institutions to comply with in their daily practice, thereby protecting patients, trainees and physicians. We believe that our study outcomes have implications for such policies.

Our study also has several limitations. First, only 19.8% of the surveyed students had been in clinical rotation for more than 1 year. As the time of clinical exposure may influence views on the issue of identity disclosure, our results may be less representative for senior students. Second, we investigated the identity dilemma only from the perspective of medical students but lacked data and comments from supervising physicians and patients. Third, we could demonstrate only an association between the ambiguous introduction of identity and more “adverse events” but could not prove a causal relationship. “Adverse events” were also perhaps not the optimal way to evaluate the means of introduction, as distrust was somewhat subjective to define and medical conflict events occurred relatively rarely overall. Other studies have investigated the issue using “quality of care” as the measurement evaluated by patients [18,35]. We call on further studies to combine these data to reveal the full extent of the issue. Finally, whether such issues are culture dependent and how they may differ across nations has not yet been addressed, and thus, further studies on this topic are needed.

Bias, misunderstandings, rejection and even fear of the medical students from the public may be rooted in their concern for health; however, successful integration of medical students into a clinical setting while maintaining patient satisfaction and perceived value of care can be achieved [36]. Given that the professional development of these doctors-to-be, which heavily relies on bedside learning, is of great public interest, the successful resolution of the identity disclosure “dilemma” and the learning opportunities it engenders deserves collaborative efforts from all the involved parties.

## Conclusions

It is beneficial for medical students to clearly explain their identity to patients in order to decrease patient distrust and refusal to perform procedures. Several methods to help resolve the ethical dilemma of identity disclosure include emphasizing the role of mentors and the importance of faculty development, developing educational curriculum concentrating on both traditional medical ethics and cultivating moral resilience for medical students, and creating clear regulations and guidelines for disclosing the identity of medical students.

## Supporting information

S1 File Questionnaire. The translated English version of the questionnaire on the ethical dilemma of identity faced by medical students in clinical practices among teaching hospitals affiliated to 13 medical schools in China.
(DOCX)

S1 Dataset Raw data. Participants’ answers to the questionnaire.
(XLS)

S1 Table Entire results. Results of Part II of the questionnaire addressing present situation and students’ perception of the identity issue of medical students in clinical practice.
(DOCX)
