# Supplementary material for: Ethical dilemma of identity disclosure faced by medical students in clinical clerkships: A nationwide multicenter study in China
Source: PLoS One. 2018 Jul 11;13(7):e0200335. doi: 10.1371/journal.pone.0200335 (PMC6040732; doi:10.1371/journal.pone.0200335)
Supplement: S1 File — The translated English version of the questionnaire on the ethical dilemma of identity faced by medical students in clinical practices among teaching hospitals affiliated to 13 medical schools in China. (DOCX) [file pone.0200335.s001.docx]

**Questionnaire**

**The translated English version of the questionnaire on the ethical dilemma of identity faced by medical students in clinical practices among teaching hospitals affiliated to 13 medical schools in China.**

**Part I**

1. Your gender:
2. Male
3. Female
4. Your medical school
5. Peking Union Medical College
6. Peking University Health Science Center
7. Xiangya School of Medicine, Central South University
8. Tongji Medical College, Huazhong University of Science and Technology
9. Southern Medical University
10. Shanghai Medical College of Fudan University
11. West China Medical Center, Sichuang University
12. Medical College of Soochow University
13. Wuhan University School of Medicine
14. China Medical University
15. Shanghai Jiao Tong University School of Medicine
16. Zhejiang University School of Medicine
17. Zhongshan School of Medicine
18. How long have you been directly involved in direct patient care?
19. Less than 6 months
20. 6 months to 1 year
21. More than 1 year
22. Was clinical medicine your first choice when applying for college?
23. Yes
24. No
25. Do you have any family members who are also health care workers?
26. Yes
27. No
28. Have you ever been hospitalized or have you ever taken care of someone who was hospitalized?
29. Yes
30. No

**Part II**

1. When your preceptors introduce you to the patients and their families, how would he/she address you?
2. He/she would introduce you as student and integral part of the team (e.g. to introduce you with a statement like “(W)e have a medical student in our team”)
3. He/she would call you a doctor (e.g. to introduce you with a statement like “(T)his is Dr. …”)
4. If a particular clinical practice is mainly managed by medical students like you, how would you deal with your identity?

A．I would be honest about my role as a medical student (e.g. to introduce yourself with a statement like “I am a medical student in the team”)

B．I would be ambiguous about my identity or not explicitly tell unless asked (e.g. to introduce yourself with a statement like “I am Dr. …”)

1. What do you think is the biggest problem if the patients know your identity as a medical student?
2. The patients will not cooperate in the diagnosis and treating process
3. The patients will not cooperate with in the teaching process
4. It will negatively affect the doctor-patient relationship
5. It makes no difference whether the patients know your identity or not
6. It will benefit both parties
7. It will cause unnecessary trouble to medical students
8. How many times have you been distrusted because of your identity in your clinical working experience (e.g. patients or patients’ relatives showed distrust towards you verbally) in the past 6 months?
9. Never
10. Once or twice
11. More than twice
12. How many times have you been refused to perform medical procedures by the patients because of your identity in the past 6 months?
13. Never
14. Once or twice
15. More than twice
16. How many times have you been involved in a medical conflict with patients (e.g. patients threatened to sue you or your institution for malpractice) due to your identity in the past 6 months?
17. Never
18. Once or twice
19. More than twice
20. Do you think it is necessary to disclose your identity in the following procedures?
    1. Taking medical history & conducting physical examination
    2. Noninvasive medical procedures like dressing changes
    3. Low-risk invasive medical procedures like venous blood draws
    4. High-risk invasive medical procedures like bone marrow aspiration and lumbar puncture
21. How do you feel about patients' distrust towards medical students?
22. It is reasonable and justified
23. It is not fully justified but still understandable
24. It is totally unjustified and biased
25. Do you feel that the patients have every right to know the true identities of all team members involved in the medical care?
26. Yes
27. No
28. How do you think divulging true identities of all staff members to patients will affect care?
    1. It will hinder normal care
    2. It will negatively affect development of a young doctor
    3. It will negatively affect the doctor-patient relationships
    4. It will not bear serious negative effects
29. Do you think that the school should train preceptors to deal with students’ identity issue?
30. Yes
31. No
32. Do you think that the patients realize that it is a routine for medical students to participate in medical care in teaching hospitals?
33. Yes
34. No
35. Which do you think is the best way to resolve stigma around identity issues of medical students?
36. The government should introduce policies regulating medical students' rights and obligations
37. The hospital should reinforce the role of teaching hospitals to patients
38. Social media platforms should help clarify misunderstandings between patients and medical students
39. I do not believe it is an issue
40. Others
41. Your free comments on this ethical dilemma of identity:

____________________________________________________________
