# Supplementary material for: Ethical dilemma of identity disclosure faced by medical students in clinical clerkships: A nationwide multicenter study in China
Source: PLoS One. 2018 Jul 11;13(7):e0200335. doi: 10.1371/journal.pone.0200335 (PMC6040732; doi:10.1371/journal.pone.0200335)
Supplement: S1 Table — Results of Part II of the questionnaire addressing present situation and students’ perception of the identity issue of medical students in clinical practice. (DOCX) [file pone.0200335.s003.docx]

**Entire results**

**Results of Part II of the questionnaire addressing present situation and students’ perception of the identity issue of medical students in clinical practice.**

| Questions | Number(%) |
| --- | --- |
| When your preceptors introduce you to the patients and their families, how would he/she address you? | |
| A. He/she would introduce you as student and integral part of the team (e.g. to introduce you with a statement like “(W)e have a medical student in our team”) | 559(59.0%) |
| B. He/she would call you a doctor (e.g. to introduce you with a statement like “(T)his is Dr. …”) | 388(41.0%) |
| If a particular clinical practice is mainly managed by medical students like you, how would you deal with your identity? | |
| A. I would be honest about my role as a medical student (e.g. to introduce yourself with a statement like “I am a medical student in the team”) | 271(28.6%) |
| B. I would be ambiguous about my identity or not explicitly tell unless asked (e.g. to introduce yourself with a statement like “I am Dr. …”) | 676(71.4%) |
| What do you think is the biggest problem if the patients know your identity as a medical student? | |
| A. The patients will not cooperate in the diagnosis and treating process | 351(37.1%) |
| B. The patients will not cooperate with in the teaching process | 182(19.2%) |
| C. It will negatively affect the doctor-patient relationship | 126(13.3%) |
| D. It makes no difference whether the patients know your identity or not | 59(6.2%) |
| E. It will benefit both parties | 111(11.7%) |
| F. It will cause unnecessary trouble to medical students | 118(12.5%) |
| How many times have you been distrusted because of your identity in your clinical working experience (e.g. patients or patients’ relatives showed distrust towards you verbally) in the past 6 months? | |
| A. Never | 375(39.6%) |
| B. Once or twice | 382(40.3%) |
| C. More than twice | 190(20.1%) |
| How many times have you been refused to perform medical procedures by the patients because of your identity in the past 6 months? | |
| A. Never | 573(60.5%) |
| B. Once or twice | 273(28.8%) |
| C. More than twice | 101(10.7%) |
| How many times have you been involved in a medical conflict with patients (e.g. patients threatened to sue you or your institution for malpractice) due to your identity in the past 6 months? | |
| A. Never | 850(89.8%) |
| B. Once or twice | 77(8.1%) |
| C. More than twice | 20(2.1%) |
| Do you think it is necessary to disclose your identity in the following procedures? | |
| Taking medical history & conducting physical examination | |
| A. Yes | 169(17.8%) |
| B. No | 778(82.2%) |
| Noninvasive medical procedures like dressing change | |
| A. Yes | 145(15.3%) |
| B. No | 802(84.7%) |
| Low-risk invasive medical procedures like venous blood drawing | |
| A. Yes | 341(36.0%) |
| B. No | 606(64.0%) |
| High-risk invasive medical procedures like bone marrow aspiration and lumbar puncture | |
| A. Yes | 716(75.6%) |
| B. No | 231(24.4%) |
| How do you feel about patients' distrust towards medical students? | |
| A. It’s reasonable and justified | 201(21.2%) |
| B. It’s not fully justified but still understandable | 719(75.9%) |
| C. It’s totally unjustified and biased | 27(2.9%) |
| Do you feel that the patients have every right to know the true identities of all team members involved in the medical care? | |
| A. Yes | 854(90.2%) |
| B. No | 93(9.8%) |
| How do you think divulging true identities of all staff members to patients will affect care? | |
| It will hinder normal arrange |  |
| A. Yes | 430(45.4%) |
| B. No | 517(54.6%) |
| It will negatively affect development of a young doctor | |
| A. Yes | 617(65.2%) |
| B. No | 330(34.8%) |
| It will negatively affect the doctor-patient relationships | |
| A. Yes | 308(32.5%) |
| B. No | 639(67.5%) |
| It will not bear serious negative effects | |
| A. Yes | 138(14.6%) |
| B. No | 809(85.4%) |
| Do you think that the school should train preceptors to deal with students’ identity issue? | |
| A. Yes | 852(90.0%) |
| B. No | 95(10.0%) |
| Do you think that the patients realize that it is a routine for medical students to participate in medical care in teaching hospitals? | |
| A. Yes | 569(60.1%) |
| B. No | 378(39.9%) |
| Which do you think is the best way to resolve stigma around identity issues of medical students? | |
| A. The government should introduce policies regulating medical students' rights and obligations | 539(56.9%) |
| B. The hospital should reinforce the role of teaching hospitals to patients | 109(11.5%) |
| C. Social media platforms should help clarify misunderstandings between patients and medical students | 242(25.6%) |
| D. I do not believe it is an issue | 38(4.0%) |
| E. Others | 19(2.0%) |
